# Supplementary material for: A linear response framework for quantum simulation of bosonic and fermionic correlation functions
Source: Nat Commun. 2024 May 8;15:3881. doi: 10.1038/s41467-024-47729-z (PMC11079044; doi:10.1038/s41467-024-47729-z)
Supplement: Supplementary file 1 — Supplementary Information [file 41467_2024_47729_MOESM1_ESM.pdf]

Supplementary Information

Efekan Kökcü [1,*] Heba A. Labib [1] J. K. Freericks [2] and A. F. Kemper [1,†]
[1] *Department of Physics, North Carolina State University, Raleigh, North Carolina 27695, USA*
[2] *Department of Physics, Georgetown University, 37th and O Sts. NW, Washington, DC 20057 USA*
(Dated: March 28, 2024)

Supplementary Note 1. Raw data and analysis for the electronic Green's function

In this section, we provide the full data for $\mathcal{L}_k(t)$ and $|\mathcal{L}_k(\omega)|^2$ obtained via momentum selective linear response applied on SSH model (the data can also be found at https://datadryad.org/stash/share/Gml-JU8szrzZtFsWWS_BNcOF6eoXOXbYHXZVAFo7goI). While the data in the bottom row is shown in Fig. 2 as false color plots, here we provide line plots of the same data for clarity. For each k and δ we collected 3 data sets with 8,000 shots each, yielding 24,000 shots total per curve. As discussed in the main text, $\mu = 5$, $V_{nn} = 1$ and the amplitude of the signal $\eta\Delta t = 0.04$. While obtaining the data we incorporated dynamical decoupling and Pauli twirling as implemented in the *qiskit_research* package, and did not apply any measurement error mitigation method.

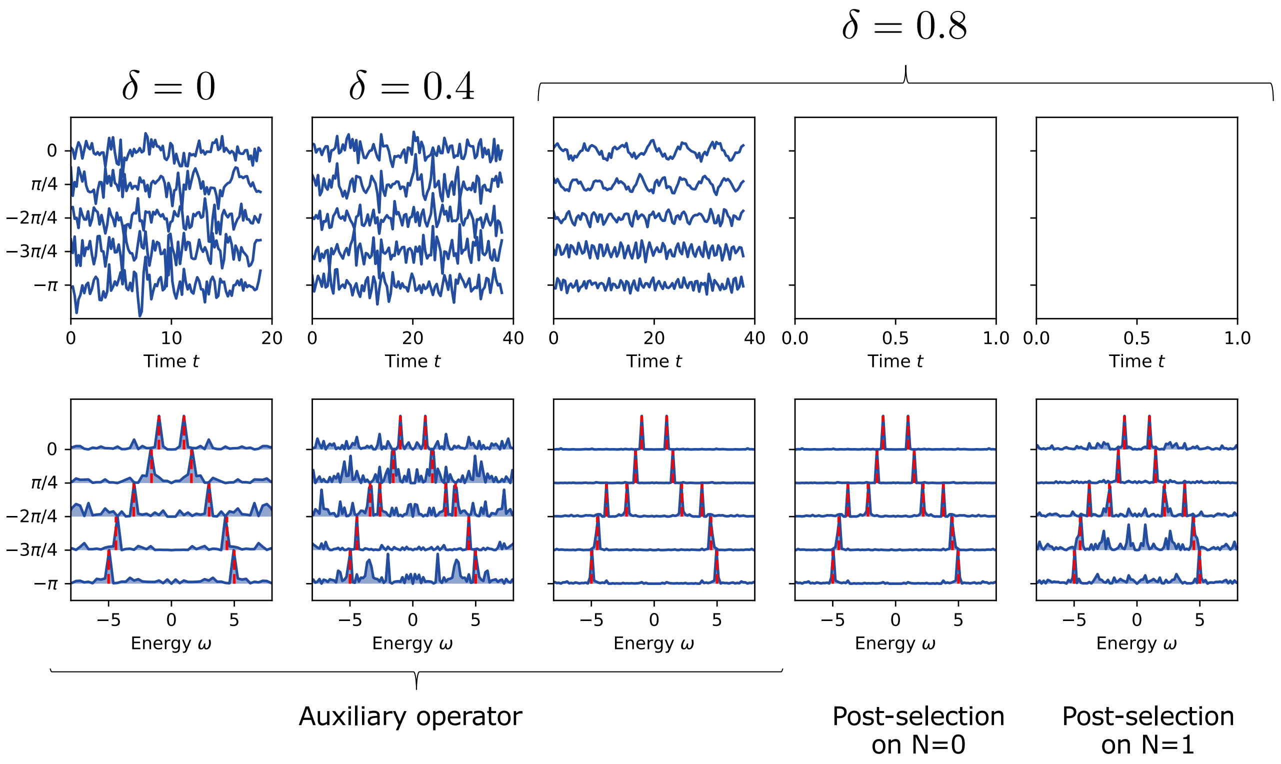

Supplementary Figure 1. Data from *ibm_auckland* for the three values of δ presented in the main text, as well as the corresponding $|\mathcal{L}_k(\omega)|^2$. Vertical red dashed lines indicate the expected energies. As in the main text, $\mu = 5$ and $V_{nn} = 1$. Note that two copies of the Green's function appear at positive and negative energies (see text for discussion).

* ekokcu@ncsu.edu
† akemper@ncsu.edu

Supplementary Note 2. Raw data and analysis for the comparison of momentum-selective linear response, real space linear response, and Hadamard test

In order to make a comparison between the linear response method in real and momentum space as well as the Hadamard test method, we performed noisy simulations for each. We constructed a noise model by adding adjustable quantum errors to single and multi qubits gates. The model mainly depends on adding depolarizing quantum channels that mainly decohere qubits; the decoherence is either a result of phase flip or a bit flip or both. We added a fixed single-qubit depolarizing error with a 0.1% rate and a 2-qubit depolarizing error once with a 10% rate and once with a 20% rate. In performing the calculations, we have forced the noisy simulator to respect the linear connectivity found on IBM quantum computers.

The results of the simulations, which are $\mathcal{L}_k(t)$ for the momentum-selective linear response and $\mathcal{L}(r,t)$ for the others, are shown in Supplementary Fig. 2. The latter two are Fourier transformed to $\mathcal{L}_k(t)$ as well, and all three are further transformed to $\mathcal{L}_k(\omega)$. As discussed in the main text, and as is clear from the both the line and false-color plots of $|\mathcal{L}_k(\omega)|$, the momentum-selective linear response method outperforms the other two in terms of signal to noise ratio.

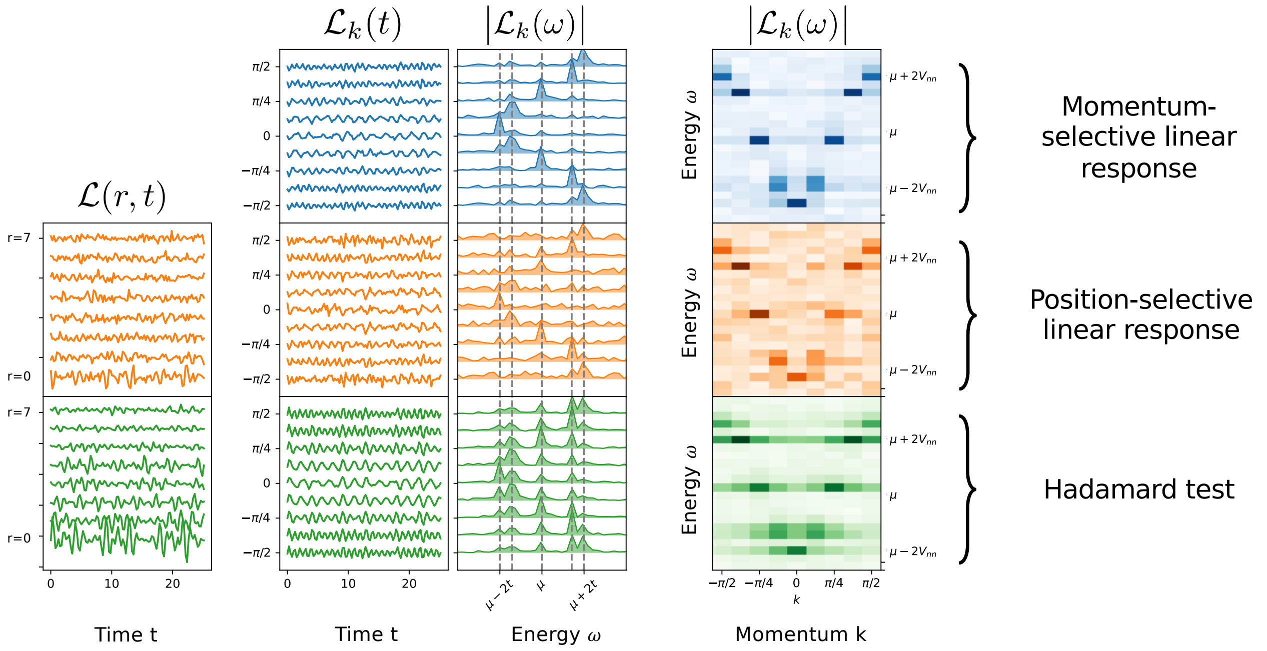

Supplementary Figure 2. **Left:** Noisy simulator data of $\mathcal{L}(r,t)$. Note that the momentum-selective method avoids this step. **Center:** momentum-space Green's function as a function of time t or frequency ω. **Right:** false-color plot of $|\mathcal{L}_k(\omega)|$.

A. Noise analysis for the Fourier transform of Hadamard test results

These results can be analyzed by approximating the noise as a depolarizing noise coming from swap gates used in the implementation of the driving field (or correlation function operators in the Hadamard test), and Gaussian noise caused by the shot noise. For this analysis, we assume that the time evolution is done with no noise. In this case, the momentum selective linear response method only has shot noise, where each $\mathcal{L}_k(\omega)$ is measured with the same noise. Similarly, the position selective linear response, only has shot noise, which is Fourier transformed to momentum space. Because the shot noise is not correlated, the noise in the Fourier space remains uncorrelated.

For the Hadamard test, however, in order to calculate $G_{ij}(t) = G_{0,i-j}(t) = G_{i-j}(t)$, one needs to apply $i-j-1$ swap gates if the Hardware topology is linear. This changes the depolarization noise structure since swap gates are made of 3 CNOT gates. We can assume that for each swap gate, with a small probability p we obtain a completely polarized state, and we get the correct state otherwise. It can be shown that this merely dampens the signal, which leads to

$$G^M_r(t) = (1-p)^r G_r(t) \tag{1}$$

where $G_r^M(t)$ is the measured Green's function and $G_r(t)$ is the exact one.

More generally, we can assume the existence of position-structured noise $f(r)$, and write that $G_r^M(t) = f(r)G_r(t)$. In this case, when we apply Fourier transform, we get (for n sites)

$$G_k^M(t) = \frac{1}{\sqrt{n}} \sum_r e^{-ikr} f(r) G_r(t) = \frac{1}{n} \sum_{r,p} e^{i(p-k)r} f(r) G_p(t) = \frac{1}{\sqrt{n}} \sum_p f_{k-p} G_p(t). \tag{2}$$

Thus, the measured Green's function has contribution from other momentum modes.

The second contribution comes from the shot noise, which arises for both position-basis measurements (linear response and Hadamard test). When Fourier transforming from real to momentum space, the measurement error (variance) from the shot noise leads to a corresponding error in the Fourier transform as

$$\Delta G_k^2 = \frac{1}{n} \sum_r \cos(kr)^2 \Delta G_r^2 \tag{3}$$

On average, this will lead to $\Delta G_k = \frac{1}{\sqrt{2}} \Delta G_r$. Now, the prefactor of n appears in the signal as well as the noise, and the operative quantity is the signal-to-noise ratio $\Delta G_k / G_k$, which scales as $\sqrt{n}$ with the system size. The underlying reason for this is simply we are adding n variables with the same variance (same number of shots, same measurement). This is why momentum selective linear response had better signal-to-noise than the position selective linear response in calculating G_k.

Supplementary Note 3. Derivation for obtaining $G^>$ and $G^<$ via post-selection

A. Post selection for a particle conserving Hamiltoniain for an N-particle initial state

We will demonstrate that the lesser (occupied) and greater (unoccupied) Green's functions can be directly obtained from the measurements by post-selecting on the particle number. In order to do so, we will recast the circuit calculation in fermionic language. Starting from an N-particle state $|\Psi\rangle$, we apply the momentum creation operator $\mathcal{K} = \exp(-i2\eta \sum_m \alpha_m \tilde{X}_m)$ where $\tilde{X}_m = Z_1 ... Z_{m-1} X_m$ which is equal to $(1/2)\left(c_m + c_m^\dagger\right)$ after a Jordan Wigner transformation, to find (to first order in η),

$$\mathcal{K} |\Psi\rangle \approx |\Psi\rangle - i\eta\alpha_m c_m |\Psi\rangle - i\eta\alpha_m c_m^\dagger |\Psi\rangle . \tag{4}$$

Moreover, for notational clarity we have suppressed internal sums over m by using Einstein summation convention . We next apply the time evolution operator $\mathcal{U}$, and since we will be measuring the 1st qubit in the X basis, we rotate it by about y,

$$R_{1y}(\pi/4) = \frac{1}{\sqrt{2}} \left(1 + c_1^\dagger - c_1\right). \tag{5}$$

Applying this to Supplementary Eq. (4), we find

$$\begin{aligned} |\Phi^y\rangle := R_{1y}(\pi/4)\mathcal{U}\mathcal{K} |\Psi\rangle = &\frac{1}{\sqrt{2}} \left(\mathcal{U} - i\eta\alpha_m \mathcal{U} c_m - i\eta\alpha_m \mathcal{U} c_m^\dagger\right) |\Psi\rangle \\ &+ \frac{1}{\sqrt{2}} \left(c_1^\dagger \mathcal{U} - i\eta\alpha_m c_1^\dagger \mathcal{U} c_m - i\eta\alpha_m c_1^\dagger \mathcal{U} c_m^\dagger\right) |\Psi\rangle \\ &- \frac{1}{\sqrt{2}} \left(c_1 \mathcal{U} - i\eta\alpha_m c_1 \mathcal{U} c_m - i\eta\alpha_m c_1 \mathcal{U} c_m^\dagger\right) |\Psi\rangle . \end{aligned} \tag{6}$$

At this point, we can read off the particle number for each term. Since $|\Psi\rangle$ has N particles and the Hamiltonian is particle conserving, counting the number of annihilation and creation operators we see that the resulting state is a

superposition of $N-2, N-1, N, N+1$ and $N+2$ particle states. These states are

$$\begin{aligned}
|\Phi^y_{N-2}\rangle &= i\frac{\eta}{\sqrt{2}}\alpha_m c_1 \mathcal{U} c_m \,|\Psi\rangle \,, \\
|\Phi^y_{N-1}\rangle &= \frac{1}{\sqrt{2}}\Big(- c_1\mathcal{U} - i\eta\alpha_m \mathcal{U} c_m\Big)\,|\Psi\rangle \,, \\
|\Phi^y_{N}\rangle &= \frac{1}{\sqrt{2}}\Big(\mathcal{U} + i\eta\alpha_m c_1\mathcal{U} c_m^\dagger - i\eta\alpha_m c_1^\dagger \mathcal{U} c_m\Big)\,|\Psi\rangle \,, \\
|\Phi^y_{N+1}\rangle &= \frac{1}{\sqrt{2}}\Big(c_1^\dagger\mathcal{U} - i\eta\alpha_m \mathcal{U} c_m^\dagger\Big)\,|\Psi\rangle \,, \\
|\Phi^y_{N+2}\rangle &= -i\frac{\eta}{\sqrt{2}}\alpha_m c_1^\dagger \mathcal{U} c_m^\dagger \,|\Psi\rangle \,.
\end{aligned} \tag{7}$$

We will be measuring expectation values with these states. It will be mainly their norms and expectation value of $c_1^\dagger c_1$ which can be obtained via Z_1 measurement. Observing that $|\Phi_{N\pm2}\rangle \sim \eta$, they will not contribute up to linear order in η. Independent quantities to linear order in η are

$$\begin{aligned}
\langle\Phi^y_{N-1}|\Phi^y_{N-1}\rangle &= \frac{1}{2}\,\langle n_1(t)\rangle + \frac{i\eta\alpha_m}{2}\,\Big(\langle c_1^\dagger(t) c_m\rangle - \langle c_m^\dagger c_1(t)\rangle\Big) = \frac{1}{2}\,\langle n_1(t)\rangle - \eta\alpha_m \mathrm{Re}\, G^<_{1m}(t) \\
\langle\Phi^y_{N+1}|\Phi^y_{N+1}\rangle &= \frac{1}{2} - \frac{1}{2}\,\langle n_1(t)\rangle + \frac{i\eta\alpha_m}{2}\,\Big(\langle c_m c_1^\dagger(t)\rangle - \langle c_1(t) c_m^\dagger\rangle\Big) = \frac{1}{2} - \frac{1}{2}\,\langle n_1(t)\rangle + \eta\alpha_m \mathrm{Re}\, G^>_{1m}(t) \\
\langle\Phi^y_{N}|c_1^\dagger c_1|\Phi^y_{N}\rangle &= \frac{1}{2}\,\langle n_1(t)\rangle + \frac{i\eta\alpha_m}{2}\,\Big(\langle c_m^\dagger c_1(t)\rangle - \langle c_1^\dagger(t) c_m\rangle\Big) = \frac{1}{2}\,\langle n_1(t)\rangle + \eta\alpha_m \mathrm{Re}\, G^<_{1m}(t)
\end{aligned} \tag{8}$$

This leads to first two equations of Eq.12. Instead, if we apply a rotation around x we get:

$$R_{1x}(\pi/4) = \frac{1}{\sqrt{2}}\,\Big(1 + ic_1^\dagger + ic_1\Big)\,, \tag{9}$$

then

$$\begin{aligned}
|\Phi^x\rangle := R_{1x}(\pi/4)\mathcal{U}\mathcal{K}\,|\Psi\rangle = \frac{1}{\sqrt{2}}&\left(\mathcal{U} - i\eta\alpha_m\mathcal{U}c_m - i\eta\alpha_m \mathcal{U} c_m^\dagger\right)|\Psi\rangle \\
+\frac{i}{\sqrt{2}}&\left(c_1^\dagger\mathcal{U} - i\eta\alpha_m c_1^\dagger\mathcal{U}c_m - i\eta\alpha_m c_1^\dagger\mathcal{U} c_m^\dagger\right)|\Psi\rangle \\
+\frac{i}{\sqrt{2}}&\left(c_1\mathcal{U} - i\eta\alpha_m c_1\mathcal{U}c_m - i\eta\alpha_m c_1\mathcal{U} c_m^\dagger\right)|\Psi\rangle \,.
\end{aligned} \tag{10}$$

Then, the components with different particle number are

$$|\Phi^x_{N-2}\rangle = \frac{\eta}{\sqrt{2}}\alpha_m c_1\mathcal{U}c_m\,|\Psi\rangle \,, \tag{11a}$$

$$|\Phi^x_{N-1}\rangle = \frac{1}{\sqrt{2}}\Big(ic_1\mathcal{U} - i\eta\alpha_m\mathcal{U}c_m\Big)\,|\Psi\rangle \,, \tag{11b}$$

$$|\Phi^x_{N}\rangle = \frac{1}{\sqrt{2}}\Big(\mathcal{U} + \eta\alpha_m c_1\mathcal{U}c_m^\dagger + \eta\alpha_m c_1^\dagger\mathcal{U}c_m\Big)\,|\Psi\rangle \,, \tag{11c}$$

$$|\Phi^x_{N+1}\rangle = \frac{1}{\sqrt{2}}\Big(ic_1^\dagger\mathcal{U} - i\eta\alpha_m\mathcal{U}c_m^\dagger\Big)\,|\Psi\rangle \,, \tag{11d}$$

$$|\Phi^x_{N+2}\rangle = \frac{\eta}{\sqrt{2}}\alpha_m c_1^\dagger\mathcal{U}c_m^\dagger\,|\Psi\rangle \,. \tag{11e}$$

To linear order in η, independent quantities that can be derived from norms and the expectation value of Z_1 are

$$\begin{aligned}
\langle \Phi^x_{N-1} | \Phi^x_{N-1} \rangle &= \frac{1}{2} \langle n_1(t) \rangle - \frac{\eta \alpha_m}{2} \left(\langle c_1^\dagger(t) c_m \rangle + \langle c_m^\dagger c_1(t) \rangle \right) \\
&= \frac{1}{2} \langle n_1(t) \rangle - \eta \alpha_m \mathrm{Im}\, G^<_{1m}(t) \\
\langle \Phi^x_{N+1} | \Phi^x_{N+1} \rangle &= \frac{1}{2} - \frac{1}{2} \langle n_1(t) \rangle - \frac{\eta \alpha_m}{2} \left(\langle c_m c_1^\dagger(t) \rangle + \langle c_1(t) c_m^\dagger \rangle \right) \\
&= \frac{1}{2} - \frac{1}{2} \langle n_1(t) \rangle + \eta \alpha_m \mathrm{Im}\, G^>_{1m}(t) \\
\langle \Phi^x_N | c_1^\dagger c_1 | \Phi^x_N \rangle &= \frac{1}{2} \langle n_1(t) \rangle + \frac{\eta \alpha_m}{2} \left(\langle c_m^\dagger c_1(t) \rangle + \langle c_1^\dagger(t) c_m \rangle \right) \\
&= \frac{1}{2} \langle n_1(t) \rangle + \eta \alpha_m \mathrm{Im}\, G^<_{1m}(t)
\end{aligned}$$

These can be linearly combined to obtain the final two equations of Eq.12 in the main text, and shows that retarded, lesser and greater fermionic Green's functions can be calculated via post selection.

B. Post selection for SSH model for 0-particle initial state

In this case our calculation simplifies drastically, since we cannot annihilate a particle from a 0-particle state, and thus the only contribution will come from 0, 1 and 2 particle states:

$$\begin{aligned}
|\Phi^y_0\rangle &= \frac{1}{\sqrt{2}} \left(\mathcal{U} + i\eta\alpha_m c_1 \mathcal{U} c_m^\dagger - i\eta\alpha_m c_1^\dagger \mathcal{U} c_m \right) |0\rangle \\
&= \frac{1}{\sqrt{2}} \left(\mathcal{U} + i\eta\alpha_m c_1 \mathcal{U} c_m^\dagger \right) |0\rangle \,, \\
|\Phi^y_1\rangle &= \frac{1}{\sqrt{2}} \left(c_1^\dagger \mathcal{U} - i\eta\alpha_m \mathcal{U} c_m^\dagger \right) |0\rangle \,, \\
|\Phi^y_2\rangle &= -i \frac{\eta}{\sqrt{2}} \alpha_m c_1^\dagger \mathcal{U} c_m^\dagger |0\rangle \,.
\end{aligned} \tag{12}$$

The norm of the 2-particle contribution is $O(\eta^2)$ and is neglected. The norms of 0- and 1-particle contributions are

$$\begin{aligned}
\langle \Phi^x_0 | \Phi^y_0 \rangle &= \frac{1}{2} - \eta\alpha_m \mathrm{Re}\, G^>_{1m}(t), \\
\langle \Phi^x_1 | \Phi^y_1 \rangle &= \frac{1}{2} + \eta\alpha_m \mathrm{Re}\, G^>_{1m}(t).
\end{aligned}$$

Because the lesser Green's function of the 0-particle state $|0\rangle$ is zero, we can replace the greater Green's functions with the retarded ones:

$$\begin{aligned}
\langle \Phi^x_0 | \Phi^y_0 \rangle &= \frac{1}{2} - \eta\alpha_m \mathrm{Re}\, G^R_{1m}(t), \\
\langle \Phi^x_1 | \Phi^y_1 \rangle &= \frac{1}{2} + \eta\alpha_m \mathrm{Re}\, G^R_{1m}(t),
\end{aligned}$$

and therefore these partial norms contain information about the single-particle energy spectrum.

Supplementary Note 4. Quantum circuit for the SSH Model Green's function

The circuit in Fig. 2**b** mainly consists of three parts: the applied field $\mathbf{B}h(t)$, the time evolution and the measurement of $\mathbf{A}$. Here we will discuss how to use the parity operator as an auxiliary to measure fermionic Green's functions.

A. Measurement of $\mathrm{Re}\, G_k(\omega)$

In this work, we measure the following quantity for SSH model on no particle state $|0\rangle$

$$\mathcal{L}_k(t) = -i \langle 0| \Big[X_0(t), \sum_r \cos(kr) X_r \Big] |0\rangle \,. \tag{13}$$

We will slowly change this into an expression given in terms of Green's functions, and show that $\mathcal{L}_k(t)$ contains information about the one particle spectral weight.

First observe that $\tilde{X}_r |0\rangle = X_r |0\rangle$. In addition, for $P = Z_0 Z_1 ... Z_{n-1}$, where n is the number of sites, we have $P|0\rangle = |0\rangle$, therefore

$$\begin{aligned} \mathcal{L}_k(t) &= -i \langle 0| \Big[X_0(t), \sum_r \cos(kr) \tilde{X}_r \Big] P |0\rangle \\ &= i \langle 0| \Big\{ X_0(t) P, \sum_r \cos(kr) \tilde{X}_r \Big\} |0\rangle \\ &= i \langle 0| \Big\{ X_0(t) P(t), \sum_r \cos(kr) \tilde{X}_r \Big\} |0\rangle \,. \end{aligned} \tag{14}$$

On the last line, we have used $P(t) = P$. Eq. 14 is essentially the auxiliary operator method given in the manuscript applied in reverse way to transform commutator into anti-commutator. Now $X_0 P = -iY_0 Z_1 ... Z_{n-1}$, and $Z_1 ... Z_{n-1} |0\rangle = |0\rangle$. In addition, $\tilde{Y}_0 = Y_0$, then

$$\mathcal{L}_k(t) = \langle 0| \Big\{ \tilde{Y}_0(t), \sum_r \cos(kr) \tilde{X}_r \Big\} |0\rangle \,. \tag{15}$$

Applying the Jordan-Wigner transformation to get the Fermionic operators back, let us plug in $\tilde{X}_r = c_r + c_r^\dagger$ and $\tilde{Y}_0 = i(c_0^\dagger - c_0)$:

$$\begin{aligned} \sum_r \cos(kr) \tilde{X}_r &= \frac{1}{2} \sum_r (e^{ikr} + e^{-ikr})(c_r + c_r^\dagger) = \frac{\sqrt{n}}{2} (c_k + c_{-k} + c_k^\dagger + c_{-k}^\dagger) \\ \tilde{Y}_0 &= i(c_0^\dagger - c_0) = \frac{i}{\sqrt{n}} \sum_q (c_q^\dagger - c_q). \end{aligned} \tag{16}$$

With these, we obtain

$$\begin{aligned} \mathcal{L}_k(t) &= -\frac{i}{2} \langle 0| \Big\{ \sum_q (c_q(t) - c_q^\dagger(t)), (c_k + c_{-k} + c_k^\dagger + c_{-k}^\dagger) \Big\} |0\rangle \,. \\ &= -\frac{i}{2} \sum_q \Big(\langle 0| \{ c_q(t), c_k^\dagger + c_{-k}^\dagger \} |0\rangle - \langle 0| \{ c_q^\dagger(t), c_k + c_{-k} \} |0\rangle \Big). \end{aligned} \tag{17}$$

The sum can be handled directly because momentum is conserved due to translational invariance of the SSH model, then the creation/annihilation operators anticommute when momentum values are not matched. Since we assume $t > 0$, we can plug in $\theta(t) = 1$ in the definition of $G_k^R(t)$, and then obtain

$$\begin{aligned} \mathcal{L}_k(t) &= \frac{1}{2} \big(G_k^R(t) + G_{-k}^R(t) + G_k^R(t)^* + G_{-k}^R(t)^* \big) \\ &= \mathrm{Re} \big(G_k^R(t) + G_{-k}^R(t) \big). \end{aligned} \tag{18}$$

The SSH model is symmetric under spatial reflection, thus $G_k^R = G_{-k}^R$ and we get

$$\mathcal{L}_k(t) = 2\, \mathrm{Re}\, G_k^R(t) \tag{19}$$

Let us look at this in the frequency basis:

$$\begin{aligned}\mathcal{L}_k(\omega) &= \int \mathrm{d}t\, \mathcal{L}_k(t) e^{i\omega t} \\ &= 2\int \mathrm{d}t\, \mathrm{Re}\, G_k^R(t) e^{i\omega t} \\ &= \int \mathrm{d}t\, \left(G_k^R(t) + G_k^R(t)^*\right) e^{i\omega t} \\ &= G_k^R(\omega) + G_k^R(-\omega)^*\end{aligned} \tag{20}$$

Then we get

$$\begin{aligned}\mathrm{Im}\, \mathcal{L}_k(\omega) &= \mathrm{Im}\, G_k^R(\omega) - \mathrm{Im}\, G_k^R(-\omega), \\ \mathrm{Re}\, \mathcal{L}_k(\omega) &= \mathrm{Re}\, G_k^R(\omega) + \mathrm{Re}\, G_k^R(-\omega),\end{aligned} \tag{21}$$

which means that choosing $\mathbf{A}$ and $\mathbf{B}$ as given in the beginning of this subsection, we can get single particle spectral weight.

Both momentum selective and position selective methods can be used to measure $\mathcal{L}_k(t)$. The momentum selective method is to excite the state $|0\rangle$ with $\mathbf{B} = \sum_r \cos(kr) X_r$ and measuring X_0 after time evolution, which can measure $\mathcal{L}_k(t)$ with one circuit. The position selective method is to measure X_0 after exciting the state with $\mathbf{B} = X_r$ and time evolving for all $r = 1, 2, ..., n$ values.

For the data shown in Figs. 2 and 3, we only measure $\mathcal{L}_k(t)$ in linear response methods with momentum and position selectivity, to run fewer circuits. The plots show $|\mathcal{L}_k(\omega)|^2$, which is related to the retarded Green's function as

$$\begin{aligned}|\mathcal{L}_k(\omega)|^2 =& \Big(G_k^R(\omega) + G_k^R(-\omega)^*\Big)\Big(G_k^R(\omega)^* + G_k^R(-\omega)\Big), \\ =& |G_k^R(\omega)|^2 + |G_k^R(-\omega)|^2 + 2\,\mathrm{Re}\Big(G_k^R(\omega) G_k^R(-\omega)^*\Big).\end{aligned} \tag{22}$$

Because $G_k^R(\omega)$ is strongly peaked near the single-particle excitation energy, the interference term is negligible compared to the absolute squares of the terms $G_k^R(\omega)$ and $G_k^R(-\omega)$. Therefore $|\mathcal{L}_k(\omega)|^2$ contains $|G_k^R(\omega)|^2$ and its mirror image in the plots. Due to our chemical potential choice $\mu = 5$, in the positive frequencies, we only see one of these images, which gives us the information about the single particle spectrum. Supplementary Fig. 3 illustrates this point by showing that $|\mathcal{L}_k(\omega)|$ tracks the quasi-particle peaks in $|G_k^R(\omega)|$ and $\mathrm{Im}\, G_k^R(\omega)$. In the figure, we compare the real part, imaginary part and absolute value of $G_k^R(\omega)$

$$G_k^R(\omega) = \frac{1}{\omega - \omega_k + i\epsilon}, \tag{23}$$

where $\epsilon = 0.1$. As it can be seen on panel **a**, $|G^R(\omega)|$ is peaked at ω_k just as $\mathrm{Im}\, G_k^R(\omega)$, with a slighly broader peak.. Panel **b** shows that $|\mathcal{L}_k(\omega)|$ is small except at $\omega = \pm\omega_k$ energies, which are the peaks of $|G^R(\omega)|$ and $|G^R(-\omega)|$. This provides an illustration of the fact that the interference term in Supplementary Eq. 22 is indeed negligible.

B. Time Evolution Circuit

The SSH model is a free fermionic model and thus its time evolution can be compressed into a fixed depth circuit with $O(n^2)$ CNOTs and $O(n)$ depth, where n is the system size, via the algebraic compression method given in [1, 2]. The method is limited to free fermionic systems in 1D — here we use a generalization to 1D periodic systems, which will be detailed in a forthcoming publication.

For completeness, we will summarize the method for the open 1-D chain. The method relies on a structure called a "block", and is given as the following for free fermionic models (after performing the Jordan-Wigner transformation):

$$B_i(\vec{\theta}) \equiv e^{-i\theta_1\, Z_i} e^{-i\theta_2\, Z_{i+1}} e^{-i\theta_3\, X_i X_{i+1}} e^{-i\theta_4\, Y_i Y_{i+1}}\, e^{-i\theta_5\, Z_i} e^{-i\theta_6\, Z_{i+1}}. \tag{24}$$

We represent it as the diagram shown in Supplementary Fig. 4. In Ref. 1 it is proven that $B_i(\vec{\theta})$ satisfies the following properties:

1. **Fusion:** for any set of parameters $\vec{\alpha}$ and $\vec{\beta}$, there exists an $\vec{a}$ such that

$$B_i(\vec{\alpha})\, B_i(\vec{\beta}) = B_i(\vec{a}), \tag{25}$$

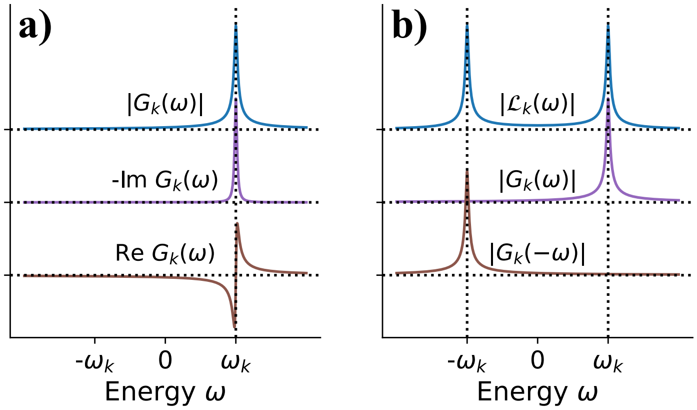

Supplementary Figure 3. Panel **a** illustrates that $|G_k^R(\omega)|$ has the same spectral behaviour as $\mathrm{Im}\, G_k^R(\omega)$, and therefore carries information about the single particle spectral weight. Panel **b** illustrates that this spectral information can be extracted from $|\mathcal{L}_k(\omega)|$ since it has two distinctive peaks, one coming from $|G_k^R(\omega)|$, the other from $|G_k^R(-\omega)|$.

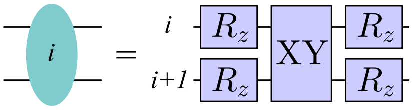

Supplementary Figure 4. Block given in (24) represented as a 2 qubit gate. XY indicates a rotation about XX followed by YY[1, 2]. R_Z, and the XX and YY rotations have independent rotation angles.

2. **Commutation:** for any set of parameters $\vec{\alpha}$ and $\vec{\beta}$, we have

$$B_i(\vec{\alpha})\, B_j(\vec{\beta}) = B_j(\vec{\beta})\, B_i(\vec{\alpha}), \qquad |i-j| > 1, \tag{26}$$

3. **Turnover:** for any set of parameters $\vec{\alpha}$, $\vec{\beta}$ and $\vec{\gamma}$ there exist $\vec{a}$, $\vec{b}$ and $\vec{c}$ such that

$$B_i(\vec{\alpha})\, B_{i+1}(\vec{\beta})\, B_i(\vec{\gamma}) = B_{i+1}(\vec{a})\, B_i(\vec{b})\, B_{i+1}(\vec{c}). \tag{27}$$

These properties can be exploited to build the triangle structure shown in Supplementary Fig. 5**a**, which can absorb any additional block by simple parameter changes. Calculation of the parameters can be done directly via linear algebra operations without any variational calculation, the details of which are given in [2].

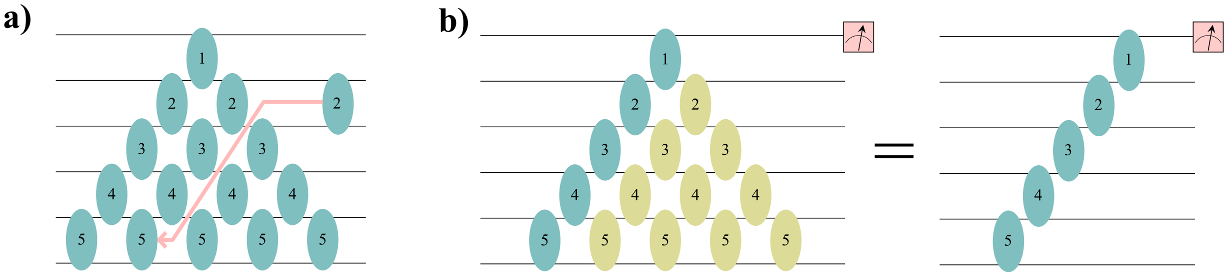

Supplementary Figure 5. Panel **a** demonstrates the triangle structure and how it can absorb a block. Block with index 2 goes down with a series of turnover operations, and ends up merging the block at the end of the arrow. Panel **b** demonstrates the additional simplification due to measurement. Blocks with gold color has no effect on the measurement on the 0th qubit, and therefore can be discarded, reducing the CNOT count from $O(n^2)$ to $O(n)$.

For the momentum selective case we only need to measure the 0th site, and thus the information on the other qubits are not relevant. As shown in Supplementary Fig. 5**b**, because the measurement is on qubit 0, blocks that do not affect qubit zero can be pushed after the measurement, and therefore can be ignored. Although post-selection

requires measurement of all qubits, this simplification can still be done simply because the only information used from the other qubits is the particle number, and the TFXY blocks do not change the particle number.

The triangle structure CNOT count is $n(n-1)/2$, which is 28 for the $n = 8$ calculations presented in the main text. After this measurement simplification, the CNOT count decreases to $2(n-1)$, or 14 for our calculations.

Supplementary Note 5. Hardware Calibration Details

The results from the quantum computer shown in Fig. 2 were run on *ibmq_auckland*, an IBM Quantum R5 Falcon system based on superconducting qubits. Calibration information for the two dates we collected data are shown in Tables Supplementary Table 1 and Supplementary Table 2 and were obtained from the Qiskit API [3]. The qubit layout is shown in Supplementary Fig. 6.

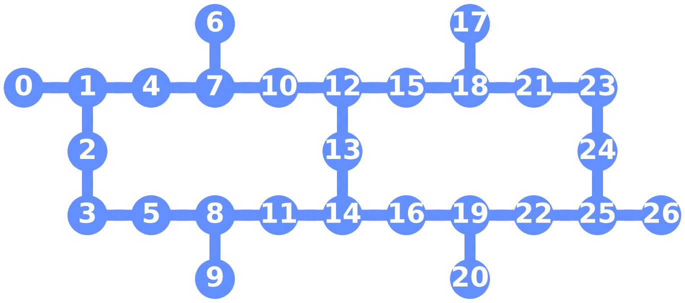

Supplementary Figure 6. Qubit layout for the IBM Quantum R5 Falcon system: *ibmq_auckland*.

Qubits	T1 (μs)	T2 (μs)	readout error (%)	CNOT connection	CNOT error (%)
13	140	27.6	0.56	13-12	0.536
12	256	232	1.84	12-10	0.835
10	225	49.2	0.91	10-7	0.544
7	130	218	0.92	7-4	0.933
4	176	164	1.85	4-1	1.08
1	52.7	135	0.95	1-2	0.594
2	173	177	1.61	2-3	0.516
3	104	67.6	1.55		

TABLE Supplementary Table 1. Calibration data for *ibmq_auckland* on September 19th, 2022.

Qubits	T1 (μs)	T2 (μs)	readout error (%)	CNOT connection	CNOT error (%)
0	372	420	0.80	0-1	0.504
1	398	285	1.02	1-2	0.763
2	528	413	0.86	2-3	0.382
3	365	131	1.29	3-5	0.340
5	253	371	21.7	5-8	0.428
8	353	109	1.67	8-11	0.371
11	146	309	1.10	11-14	0.441
14	468	129	22.9		

TABLE Supplementary Table 2. Calibration data for *ibmq_auckland* on September 30th, 2022.

Supplementary Note 6. Scaling: Constraints on the signal amplitude and Required Number of Shots for a delta pulse signal

In this section, we will talk about the possible errors and how the signal amplitude and number of shots are constrained to keep those errors smaller than a certain value of accuracy ϵ. The possible errors we analyze are non-linear contributions, Trotter error for $\exp(i\eta\mathbf{B})$ for cases that $\mathbf{B}$ is a linear combination of non-commuting terms, and the statistical error caused by the finite number of measurements i.e. shot noise.

In this analysis, we show how does the amplitude of the signal and the number of shots scale with the desired precision on the response function and certain properties of the operators $\mathbf{A}$ and $\mathbf{B}$ for a delta pulse signal $h(t) = \eta\delta(t)$. While doing this, we have assumed that the time evolution algorithm is perfect. Depending on the choice of the time evolution algorithm, the number of shots needs to be modified depending on the simulation time as well.

Results indicate that the error terms only depend on the spectral norm of the operators $||\mathbf{A}||$ and $||\mathbf{B}||$, desired precision ϵ and number of shots/sample size.

A. Nonlinear contributions

When a system is driven by $\exp(i\eta\mathbf{B})$, anything we measure will have contributions that are linear and non-linear in η. Intuitively, choosing η small enough will lead to all non-linear terms to go to zero faster than the linear term, and allow us to measure the linear response. Here we analyze how small η should be so that the non-linear contribution is smaller than some threshold value Δ.

We drive the system with $\mathbf{B}$, evolve for time t with the Hamiltonian $\mathcal{H}_0$, and then measure $\mathbf{A}$. Defining $\mathbf{A}(t) = \exp(it\mathcal{H}_0)\mathbf{A}\exp(-it\mathcal{H}_0)$, what we measure in this process is

$$A(t) = \langle\psi_0|e^{i\eta\mathbf{B}}\mathbf{A}(t)e^{-i\eta\mathbf{B}}|\psi_0\rangle = \sum_{n=0}^{\infty}\frac{(i\eta)^n}{n!}\langle\psi_0|\mathrm{ad}^n_{\mathbf{B}}\mathbf{A}(t)|\psi_0\rangle \tag{28}$$

where $\mathrm{ad}_{\mathbf{B}}\mathbf{A} := [\mathbf{B},\mathbf{A}]$. The non-linear ($n > 1$) contribution in the summation are

$$\Delta = \left|A(t) - \langle\psi_0|\mathbf{A}(t)|\psi_0\rangle - \frac{i\eta}{2}\langle\psi_0|\mathrm{ad}_{\mathbf{B}}\mathbf{A}(t)|\psi_0\rangle\right| = \left|\sum_{n=2}^{\infty}\frac{(i\eta)^n}{n!}\langle\psi_0|\mathrm{ad}^n_{\mathbf{B}}\mathbf{A}(t)|\psi_0\rangle\right|. \tag{29}$$

Now, we have

$$|\langle\psi_0|\mathrm{ad}^n_{\mathbf{B}}\mathbf{A}(t)|\psi_0\rangle| \leq (2||\mathbf{B}||)^n||\mathbf{A}||. \tag{30}$$

where $||.||$ is the spectral norm of a matrix. This is because the left hand side is a linear combination of 2^n expectation values of operators that is a product of n $\mathbf{B}$'s and 1 $\mathbf{A}$. Then,

$$\Delta \leq \sum_{n=2}^{\infty}\frac{\eta^n}{n!}|\langle\psi_0|\mathrm{ad}^n_{\mathbf{B}}\mathbf{A}(t)|\psi_0\rangle| \leq \sum_{n=2}^{\infty}\frac{\eta^n}{n!}(2||\mathbf{B}||)^n||\mathbf{A}|| = ||\mathbf{A}||\sum_{n=2}^{\infty}\frac{(2\eta||\mathbf{B}||)^n}{n!} = ||\mathbf{A}||\left(e^{2\eta||\mathbf{B}||} - 1 - 2\eta||\mathbf{B}||\right). \tag{31}$$

From the Taylor expansion of the exponential function and Taylor remainder theorem, we have

$$\left|e^x - \sum_{n=0}^{N}\frac{x^n}{n!}\right| = \left|\sum_{n=N+1}^{\infty}\frac{x^n}{n!}\right| \leq e^x\frac{x^{N+1}}{(N+1)!} \tag{32}$$

where x is a positive real number. Applying this with $N = 1$ because we are interested in the linear term, we get

$$\Delta \leq ||\mathbf{A}||\left(e^{2\eta||\mathbf{B}||} - 1 - 2\eta||\mathbf{B}||\right) \leq 2\eta^2||\mathbf{A}||\,||\mathbf{B}||^2e^{2\eta||\mathbf{B}||} \tag{33}$$

Now, assuming that $\langle\psi_0|\mathbf{A}(t)|\psi_0\rangle = 0$, this means

$$\left|\chi^R(t) - \frac{A(t)}{\eta}\right| = \frac{\Delta}{\eta} \leq 2\eta||\mathbf{A}||\,||\mathbf{B}||^2e^{2\eta||\mathbf{B}||}. \tag{34}$$

It can then be shown that for values $\epsilon_{\mathrm{NL}} \leq 2||\mathbf{A}||\,||\mathbf{B}||\log 2$ where NL stands for *non-linear*, if we choose the amplitude as $\eta = \epsilon_{\mathrm{NL}}/(4||\mathbf{A}||\,||\mathbf{B}||^{\mathbf{2}})$, then we obtain

$$\left|\chi^R(t) - \frac{A(t)}{\eta}\right| \leq \frac{\epsilon_{\mathrm{NL}}}{2}\exp\left(\frac{\epsilon_{\mathrm{NL}}}{2||\mathbf{A}||\,||\mathbf{B}||}\right) \leq \epsilon_{\mathrm{NL}}, \tag{35}$$

which means that the non-linear contribution to the response function is smaller than ϵ_{NL}.

B. Trotterization of $\exp(i\eta\mathbf{B})$

If $\mathbf{B}$ is a single Pauli string, or sum of commuting Pauli strings, then it can be applied exactly on a quantum computer. However if it contains non-commuting Pauli strings, then one way to apply $e^{i\eta\mathbf{B}}$ is the Trotter-Suzuki approximation [4]. Considering that η should be small to keep the non-linear contributions small, we will approximate the driving field by one Trotter step. With $\mathbf{B} = c_1\sigma_1 + \cdots + c_\ell\sigma_\ell$ where σ_i are Pauli strings, in [5] Theorem 1, the p-th order Trotter error to approximate the unitary $e^{i\eta\mathbf{B}}$ is stated as the following

$$||T_p(e^{-i\eta\mathbf{B}}) - e^{-i\eta\mathbf{B}}|| = \mathcal{O}\left(\tilde{\alpha}^p_{\text{comm}}(\mathbf{B})\eta^{p+1}\right), \tag{36}$$

where

$$\tilde{\alpha}^p_{\text{comm}}(\mathbf{B}) = \sum_{i_1,i_2,\ldots,i_{p+1}=1}^{\ell} |c_{i_1}\ldots c_{i_{p+1}}| \; \big|\big|\big[\sigma_{i_1},\big[\sigma_{i_2},\ldots,\big[\sigma_{i_p},\sigma_{i_{p+1}}\big]\big]\big]\big|\big|\,. \tag{37}$$

To make it more clear, for $p=1$ we get

$$\tilde{\alpha}^1_{\text{comm}}(\mathbf{B}) = \sum_{i_1,i_2=1}^{\ell} |c_{i_1}c_{i_2}| \; ||[\sigma_{i_1},\sigma_{i_2}]||\,. \tag{38}$$

In this case, the error on $A(t)$ can be found as the following

$$\begin{aligned}
|A_{\text{Trot}}(t) - A(t)| =& |\langle\psi_0|T_p(e^{i\eta\mathbf{B}})\mathbf{A}(t)T_p(e^{-i\eta\mathbf{B}})|\psi_0\rangle - \langle\psi_0|e^{i\eta\mathbf{B}}\mathbf{A}(t)e^{-i\eta\mathbf{B}}|\psi_0\rangle\,| &(39)\\
=& |\langle\psi_0|T_p(e^{i\eta\mathbf{B}})\mathbf{A}(t)T_p(e^{-i\eta\mathbf{B}}) - e^{i\eta\mathbf{B}}\mathbf{A}(t)e^{-i\eta\mathbf{B}}|\psi_0\rangle\,| &(40)\\
=& |\langle\psi_0|(T_p(e^{i\eta\mathbf{B}}) - e^{i\eta\mathbf{B}})\mathbf{A}(t)T_p(e^{-i\eta\mathbf{B}}) + e^{i\eta\mathbf{B}}\mathbf{A}(t)(T_p(e^{-i\eta\mathbf{B}}) - e^{-i\eta\mathbf{B}})|\psi_0\rangle\,| &(41)\\
\leq& \left|\langle\psi_0|(T_p(e^{i\eta\mathbf{B}}) - e^{i\eta\mathbf{B}})\mathbf{A}(t)T_p(e^{-i\eta\mathbf{B}})|\psi_0\rangle\right| + \left|\langle\psi_0|e^{i\eta\mathbf{B}}\mathbf{A}(t)(T_p(e^{-i\eta\mathbf{B}}) - e^{-i\eta\mathbf{B}})|\psi_0\rangle\right| &(42)\\
\leq& ||T_p(e^{i\eta\mathbf{B}}) - e^{i\eta\mathbf{B}}||\;||\mathbf{A}||\;||T_p(e^{i\eta\mathbf{B}})|| + ||e^{i\eta\mathbf{B}}||\;||\mathbf{A}||\;||T_p(e^{-i\eta\mathbf{B}}) - e^{-i\eta\mathbf{B}})|| &(43)
\end{aligned}$$

Since $T_p(e^{i\eta\mathbf{B}})$ and $e^{i\eta\mathbf{B}}$ are unitary, their spectral norm are equal to 1. Then we get

$$\begin{aligned}
|A_{\text{Trot}}(t) - A(t)| \leq& ||T_p(e^{i\eta\mathbf{B}}) - e^{i\eta\mathbf{B}}||\;||\mathbf{A}|| + ||\mathbf{A}||\;||T_p(e^{-i\eta\mathbf{B}}) - e^{-i\eta\mathbf{B}})|| &(44)\\
\leq& \mathcal{O}\left(\tilde{\alpha}^p_{\text{comm}}(\mathbf{B})\;||\mathbf{A}||\;\eta^{p+1}\right). &(45)
\end{aligned}$$

Specifically for $p=1$ we get

$$|A_{\text{Trot}}(t) - A(t)| \leq \mathcal{O}\left(\tilde{\alpha}^1_{\text{comm}}(\mathbf{B})\;||\mathbf{A}||\;\eta^2\right). \tag{46}$$

If we plug in what we had in the previous section for the value of η in this 1st order error, we find

$$\frac{1}{\eta}|A_{\text{Trot}}(t) - A(t)| \leq \mathcal{O}\left(\tilde{\alpha}^1_{\text{comm}}(\mathbf{B})\;||\mathbf{A}||\;\eta\right) = \mathcal{O}\left(\frac{\tilde{\alpha}^1_{\text{comm}}(\mathbf{B})}{||\mathbf{B}||^2}\epsilon_{\text{NL}}\right) \tag{47}$$

In general, we can consider r Trotter steps $T_p(e^{-i\eta/r;\mathbf{B}})^r$ to approximate $e^{-i\eta\mathbf{B}}$. In that case the error would be reduced down to the following:

$$\frac{1}{\eta}|A_{\text{Trot}}(t) - A(t)| \leq \mathcal{O}\left(\tilde{\alpha}^1_{\text{comm}}(\mathbf{B})\;||\mathbf{A}||\;\frac{\eta}{r}\right) = \mathcal{O}\left(\frac{\tilde{\alpha}^1_{\text{comm}}(\mathbf{B})}{||\mathbf{B}||^2 r}\epsilon_{\text{NL}}\right), \tag{48}$$

which gives us more control over the Trotter error. To have an idea what would those norms look like, let us examine a familiar case, namely momentum definite magnon or fermion creation. For the magnon case we have $\mathbf{B}_{\text{mag}} = \sum_{r=1}^{n}\cos(kr)X_r$, where n is the number of sites or the system size. Since each X_r commute with each other, we get $\tilde{\alpha}^1_{\text{comm}}(\mathbf{B}_{\text{mag}}) = 0$. The spectral norm of the magnon creator is $||\mathbf{B}|| = \sum_{r=1}^{n}|\cos(kr)|$ since each X_r is independent. For large system sizes n, this leads to $||\mathbf{B}|| = \mathcal{O}(n)$.

In the fermion case, $\mathbf{B}_{\text{ferm}} = \sum_{r=1}^{n}\cos(kr)\tilde{X}_r = \sqrt{n}(c_k + c_k^\dagger)$. This leads to $||\mathbf{B}_{\text{ferm}}|| = \sqrt{n}$. And since each $\tilde{X}_r$ anticommutes with each other, we get

$$\tilde{\alpha}^1_{\text{comm}}(\mathbf{B}_{\text{ferm}}) = \sum_{r,s=1}^{n}|\cos(kr)\cos(ks)| = \left(\sum_{r=1}^{n}|\cos(kr)|\right)^2 = \mathcal{O}(n^2) \tag{49}$$

This leads to a Trotter error which scales as $\mathcal{O}(n\epsilon_{\text{NL}}/r)$, which can be made independent of the system size if we choose $r = n$.

C. Statistical Error from Measurement

Another source of error is the statistical error from the measurement process. We are measuring the operator $\mathbf{A}$ after applying our signal and time evolution. This is done via repeating the circuit N_{shot} times, and simply calculating the average value of this sample. As given in [6] Eqs. 1.60-1.61, the variance of this operator and the statistical error are related as

$$\epsilon_{\text{meas}} = |A_{\text{meas}}(t) - A(t)| = \sqrt{\frac{\text{Var}(\mathbf{A})}{N_{\text{shot}}}}. \tag{50}$$

If $e^{i\eta\mathbf{B}}$ was trotterized, then we have $\epsilon_{\text{meas}} = |A_{\text{meas}}(t) - A_{\text{Trot}}(t)|$. Now the variance of the Hermitian operator $\mathbf{A}$ depends on the state, and therefore depends on our initial state $|\psi_0\rangle$. For a generic state, we know the following:

$$\text{Var}(\mathbf{A}) = \langle\mathbf{A}^2\rangle - \langle\mathbf{A}\rangle^2 \leq \langle\mathbf{A}^2\rangle \leq ||\mathbf{A}^2|| = ||\mathbf{A}||^2, \tag{51}$$

where the first equality is the definition of variance, second follows from the fact that $\mathbf{A}$ is Hermitian (therefore $\langle\mathbf{A}\rangle$ is a real number). This leads to the following upper bound

$$\epsilon_{\text{meas}} \leq \frac{||\mathbf{A}||}{\sqrt{N_{\text{shot}}}}. \tag{52}$$

This leads to the following error for the response function:

$$\frac{1}{\eta}|A_{\text{meas}}(t) - A(t)| \leq \frac{||\mathbf{A}||}{\eta\sqrt{N_{\text{shot}}}} = \frac{4||\mathbf{A}||^2||\mathbf{B}||^2}{\epsilon_{\text{NL}}\sqrt{N_{\text{shot}}}}. \tag{53}$$

D. Combined error bound

With all these, the total error can be upperbounded as the following:

$$\left|\chi^R(t) - \frac{A_{\text{meas}}(t)}{\eta}\right| \leq \left|\chi^R(t) - \frac{A(t)}{\eta}\right| + \frac{1}{\eta}|A(t) - A_{\text{Trot}}(t)| + \frac{1}{\eta}|A_{\text{Trot}}(t) - A_{\text{meas}}(t)| \tag{54}$$

$$\leq 2\eta||\mathbf{A}||\,||\mathbf{B}||^2 e^{2\eta||\mathbf{B}||} + \mathcal{O}\left(\tilde{\alpha}^1_{\text{comm}}(\mathbf{B})\,||\mathbf{A}||\,\frac{\eta}{r}\right) + \frac{||\mathbf{A}||}{\eta\sqrt{N_{\text{shot}}}}. \tag{55}$$

If we plug in our choice of η from previous subsection, the total error becomes the following

$$\left|\chi^R(t) - \frac{A_{\text{meas}}(t)}{\eta}\right| \leq \epsilon_{\text{NL}} + \mathcal{O}\left(\frac{\tilde{\alpha}^1_{\text{comm}}(\mathbf{B})}{||\mathbf{B}||^2 r}\epsilon_{\text{NL}}\right) + \frac{4||\mathbf{A}||^2||\mathbf{B}||^2}{\epsilon_{\text{NL}}\sqrt{N_{\text{shot}}}}. \tag{56}$$

For most cases, norms of the operators are $\text{Poly}(n)$ where n is the system size. Thus, to keep the error smaller than some threshold, the η should be chosen inversely polynomial with respect to n, and N_{shot} should be chosen polynomial in n, which ensures the scalability of our method.

E. Scaling for the Post Selection Method

To analyze the error, let us see the post selection method from a different perspective. In the method, we measure the state in all Z directions, and select certain bit-strings only. Another way to see this is as a measurement of a certain operator O that is diagonal in the computational basis. As an example for measuring the real part of the retarded Green's function $\text{Re}\, G^R$, we measure $\langle\Phi^x_{N-1}|\Phi^x_{N-1}\rangle + \langle\Phi^x_{N+1}|\Phi^x_{N+1}\rangle$. This can be written as the expectation value of $O^R = \text{diag}(\lambda_i)$ on state $|\Psi\rangle = |\Phi^x_{N-2}\rangle + |\Phi^x_{N-1}\rangle + |\Phi^x_N\rangle + |\Phi^x_{N+1}\rangle + |\Phi^x_{N+2}\rangle$, where λ_i is 0 if the Hamming distance of i on base 2 is different from $N-1$ and $N+1$, and it is 1 otherwise. With that and the result from [6] Eqs. 1.60-1.61, we obtain the post selection measurement error ϵ_{PS} as the following

$$\epsilon_{\text{PS}} = \sqrt{\frac{\text{Var}(O)}{N_{\text{shot}}}}. \tag{57}$$

Now, for all post selection methods we provided, we had $\mathbf{A}$ and $\mathbf{B}$ a single fermion creation operator in different basis. This leads to the fact that the diagonal operator O are either 0 or 1. This means we have $||O|| = 1$, therefore we have

$$\epsilon_{\rm PS} = \sqrt{\frac{\mathrm{Var}(O)}{N_{\rm shot}}} \leq \sqrt{\frac{||O||^2}{N_{\rm shot}}} \leq \frac{1}{\sqrt{N_{\rm shot}}}. \tag{58}$$

The error for the response functions would then be $\epsilon_{\rm PS}/\eta$, which would require a number of shots that scales quadratically with $1/\eta$, therefore polynomially with the system size and inverse polynomially with the desired precision.

Supplementary Note 7. Looseness of the error bounds

We would like to note an empirical observation about the looseness of the error bounds for $\epsilon_{\rm NL}$ and $\epsilon_{\rm Trot}$ we have provided. We will do so by considering two examples. The purpose of this section is to show that for certain problems, the desired error threshold could be achieved by orders of magnitudes less resources than the theoretical results we provided in the previous section. These observation made here regarding the bounds on Trotter error match those discussed in Refs. [7, 8].

The first example we consider it the $k = \pi$ spin-spin correlation function for the antiferromagnetic Heisenberg model with 16 sites. We would like to calculate the commutator correlation function $\langle [\mathbf{A}(t), \mathbf{B}] \rangle$ for the operators

$$\mathbf{A} = \mathbf{B} = \frac{1}{n}\left(Z_1 - Z_2 + Z_3 \pm \cdots + (-1)^{n-1} Z_n\right), \tag{59}$$

and the Hamiltonian

$$H = \sum_{i=1}^{n-1} (X_i X_{i+1} + Y_i Y_{i+1} + Z_i Z_{i+1}), \tag{60}$$

on the ground state. To investigate the effect of the non-linear terms for various η values, we calculate the correlation function exactly, and with our method by using conventional computers, and present the errors in Supplementary Fig. 7.

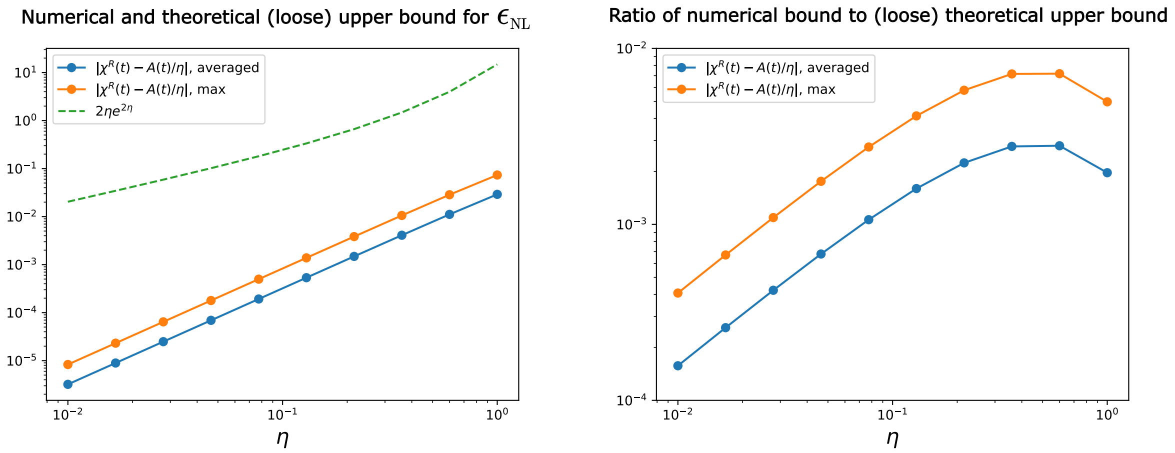

Supplementary Figure 7. Looseness of the bound on $\epsilon_{\rm NL}$. On the left we see the theoretical error bound, and the average and the maximum values of the empirical error caused by the nonlinear terms. On the right we see the ratio of the empirical errors to the theoretical error bound. As it can be seen, the actual error is 3-4 orders of magnitude smaller than the theoretical error.

In this example, the $\mathbf{B}$ operator is a linear combination of Z_i matrices, and therefore $\tilde{\alpha}^1_{\rm comm}(\mathbf{B}) = 0$ and thus incurs no Trotter error. In addition, the operators are normalized i.e. $||\mathbf{A}|| = ||\mathbf{B}|| = 1$. Then the full error given in (54) becomes $\epsilon_{\rm NL} + \epsilon_{\rm meas} = 2\eta e^{2\eta} + \frac{||\mathbf{A}||}{\eta\sqrt{N_{\rm shot}}}$. To have a total error of ϵ, one should choose η such that $\epsilon_{\rm NL} < \epsilon/2$ and $\epsilon_{\rm meas} < \epsilon/2$. The former requires η to be small enough, and the latter requires $N_{\rm shot} = 4/(\epsilon^2\eta^2)$ to be large enough. For example for $\epsilon = 0.01$, according to the data in supplementary Fig. 7, we can choose $\eta = 0.1$. Theoretical error

bound however requires us to choose $\eta = 0.00125$, which is two orders of magnitude smaller than what we need for this specific example. This would lead to a four orders of magnitude difference in the required shot count between the empirical and the theoretical non-linear errors we observe here.

The second example is momentum definite 1-particle Green's function for spinless Hubbard model with 16 sites with a momentum value $k = 2\pi/16$. To calculate the Trotter error, we apply the linear response method with and without the Trotterizing the $e^{i\eta\mathbf{B}}$ operator. We only apply one Trotter step.

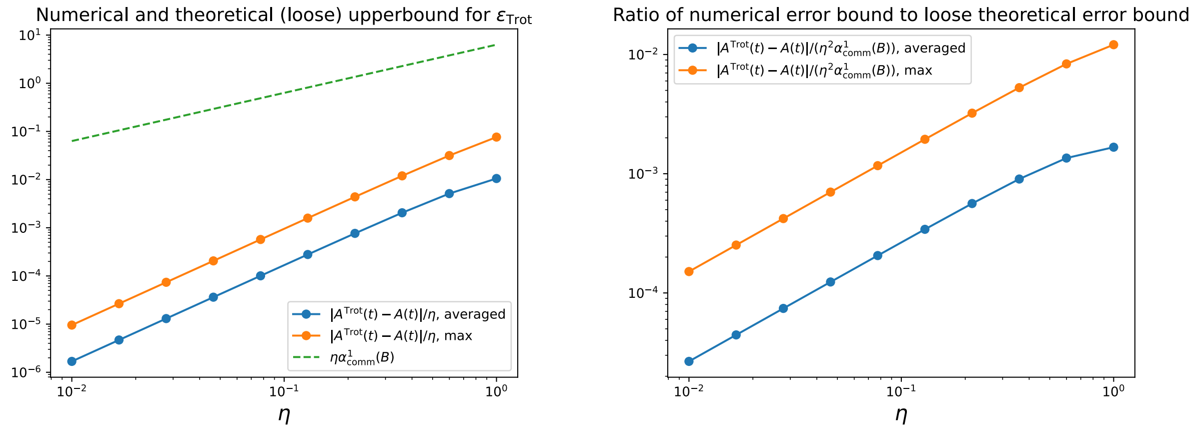

Supplementary Figure 8. Looseness of the bound on ϵ_{Trot}. On the left we see the theoretical error bound, and the average and the maximum values of the empirical error caused by Trotter approximation with a single Trotter step. On the right, we see the ratio of the average and maximum of the empirical error to the theoretical error. As it can be seen, the empirical error is 2-3 orders of magnitude smaller than the theoretical error bound for this particular example.

In this case, the operators are linear combinations of momentum definite fermion creation-annihilation operators as the following

$$\mathbf{A} = \mathbf{B} = \frac{1}{\sqrt{n}} \sum_{i=1}^{n} \cos(kr_i)\tilde{X}_i. \tag{61}$$

It can be shown that the operator norms satisfy $||\mathbf{A}|| = ||\mathbf{B}|| = 1$. However, because $\tilde{X}_i = Z_1 Z_2 \dots Z_{i-1} X_i$ do not commute with each other, the commutator norm scales linearly with the system size: $\tilde{\alpha}^1_{\mathrm{comm}}(\mathbf{B}) = \mathcal{O}(n)$. Then the theoretical Trotter error becomes $\epsilon_{\mathrm{Trot}} = n\eta/r$ where r is the number of Trotter steps. For larger system sizes, we can keep the choice of η independent of n by picking $r = n$. However, results in Supplementary Fig. 8 suggests that we do not need multiple Trotter steps to pick the η value large enough, which would lead to low number of shots. As it can be seen, for an error value of $\epsilon = 0.01$, we can pick η to be as large as 0.4, which is surprising. The theoretical choice would be $\eta = \epsilon/16 = 0.00625$, which is again two orders of magnitude smaller, and would lead to a for orders of magnitude larger number of shots.

In short, the error bounds given in the previous section are guaranteeing the fact that our method scales with system size. However, the error bounds are not tight. We observe that for certain physics-related response function calculations, the linear response method is capable of performing well even with number of shots lower, and with η values higher than the theoretical analysis.

---

[1] E. Kökcü, D. Camps, L. Bassman, J. K. Freericks, W. A. de Jong, R. Van Beeumen, and A. F. Kemper, Physical Review A **105**, 032420 (2022).
[2] D. Camps, E. Kökcü, L. Bassman, W. A. de Jong, A. F. Kemper, and R. V. Beeumen, SIAM Journal on Matrix Analysis and Applications **43**, 1084 (2022).
[3] Qiskit contributors, "Qiskit: An open-source framework for quantum computing," (2023).
[4] H. F. Trotter, Proceedings of the American Mathematical Society **10**, 545 (1959).
[5] A. M. Childs, Y. Su, M. C. Tran, N. Wiebe, and S. Zhu, Phys. Rev. X **11**, 011020 (2021).
[6] L. Lin, arXiv preprint arXiv:2201.08309 (2022).
[7] B. Şahinoğlu and R. D. Somma, npj Quantum Information **7**, 119 (2021).
[8] D. An, D. Fang, and L. Lin, Quantum **5**, 459 (2021).
